# Perfluoroalkyl substances in human bone: concentrations in bones and effects on bone cell differentiation

**DOI:** 10.1038/s41598-017-07359-6

**Published:** 2017-07-28

**Authors:** A. Koskela, J. Koponen, P. Lehenkari, M. Viluksela, M. Korkalainen, J. Tuukkanen

**Affiliations:** 1 Institute of Cancer Research and Translational Medicine, Department of Anatomy and Cell Biology, Faculty of Medicine, University of Oulu, Oulu, Finland; 2 National Institute for Health and Welfare, Chemicals and Health Unit, Kuopio, Finland; 3 Department of Environmental and Biological Sciences, University of Eastern Finland, Kuopio, Finland

## Abstract

Perfluoroalkyl substances (PFAS), including two most commonly studied compounds perfluorooctane sulfonate (PFOS) and perfluorooctanoic acid (PFOA), are widely distributed environmental pollutants, used extensively earlier. Due to their toxicological effects the use of PFAS is now regulated. Based on earlier studies on PFOA’s distribution in bone and bone marrow in mice, we investigated PFAS levels and their possible link to bone microarchitecture of human femoral bone samples (n = 18). Soft tissue and bone biopsies were also taken from a 49-year old female cadaver for PFAS analyses. We also studied how PFOA exposure affects differentiation of human osteoblasts and osteoclasts. PFAS were detectable from all dry bone and bone marrow samples, PFOS and PFOA being the most prominent. In cadaver biopsies, lungs and liver contained the highest concentrations of PFAS, whereas PFAS were absent in bone marrow. Perfluorononanoic acid (PFNA) was present in the bones, PFOA and PFOS were absent. *In vitro* results showed no disturbance in osteogenic differentiation after PFOA exposure, but in osteoclasts, lower concentrations led to increased resorption, which eventually dropped to zero after increase in PFOA concentration. In conclusion, PFAS are present in bone and have the potential to affect human bone cells partly at environmentally relevant concentrations.

## Introduction

Perfluoroalkylated substances (PFAS) have been widely used in industrial applications since the 1950s, especially as surfactants because of their stability and amphiphilic nature^1^. Their widespread use has led to wide distribution in the environment, wildlife, food and humans, especially with the most common PFAS, perfluorooctanoic acid (PFOA) and perfluorooctanesulfonic acid (PFOS)^2^ and many reports and reviews on their toxicological profiles have been published during recent years^3–5^. In addition to their persistence PFOA and PFOS have been shown to induce neonatal mortality and cause neurotoxicity and immunotoxicity among other deleterious effects^6–9^. This has led to strict regulation of PFOA and PFOS use in industrial processes, as the compounds were added to the Annex B of the Stockholm Convention on Persistent Organic Pollutants.

Earlier studies have shown that PFOS accumulates to bone and bone marrow^10, 11^. Rib bone samples taken from twenty cadavers in Catalonia, Spain, showed significant amounts of PFOA and PFOS, and the concentrations of PFOA in rib bone exceeded those in liver, lung, brain, and kidney^12^. In the adult US population, a negative association between serum PFOS and PFOA concentration and bone mineral density has been reported in cross-sectional studies^13, 14^. In our recent study, in utero and lactational exposure to PFOA in mice led to significantly higher PFOA levels in long bones even at the age of 17 months, and we showed dose-dependent effects on mouse osteoclasts and osteoblasts *in vitro* ^15^.

Bone is a highly differentiated, continuously adapting tissue consisting of osteoblasts and osteoclasts embedded in a mineralized matrix, and is vigorously modified throughout an individual’s life through a remodeling cycle consisting of bone resorption by hematopoietically derived osteoclasts and bone formation by mesenchyme-derived osteoblasts^16^. To support the human body weight, the bony skeleton has to be stiff, but also flexible enough to withstand a certain degree of deformation. Thus the optimal bone strength is composed of extrinsic and intrinsic biomechanical properties^17^. Osteoclasts and osteoblasts play key roles in maintaining bone homeostasis, and failure to adapt can lead to various bone diseases, such as osteoporosis^18^. We and others have found earlier that bone is a sensitive target of toxicity of some persistent organic pollutants, including dioxins, PCBs and tributyltin. Both adult and *in utero*/lactational exposure of rats to 2,3,7,8-tetrachlorodibenzo-*p*-dioxin (TCDD) resulted in negative impact on bone geometry, biomechanical properties and mineral density^16, 18, 19^, TCDD and tributyltin interfered with differentiation of cultured mouse osteoblasts and osteoclast showing synergistic interaction^20^, and commercial PCB mixture Aroclor 1254 inhibited differentiation of mouse osteoblastic cell line MC3T3-E1 (17)^19–23^.

These findings led us to investigate, whether PFAS can also be found in human femoral bones and how PFOA affects human osteoclasts and osteoblasts. For this purpose, we analyzed bone bank and cadaver samples and conducted *in vitro* exposure experiments with human osteoclasts and osteoblasts to see, if PFOA exposure decreases the differentiation and functioning of the cells. In order to address the toxicological significance of current human bone PFAS levels, we also studied correlations between bone and bone marrow PFAS levels and relative bone volumes. Finally, we compared human bone PFOA levels with mouse bone levels associated with adverse bone effects.

## Results

### Femoral head samples

**Figure 1:**
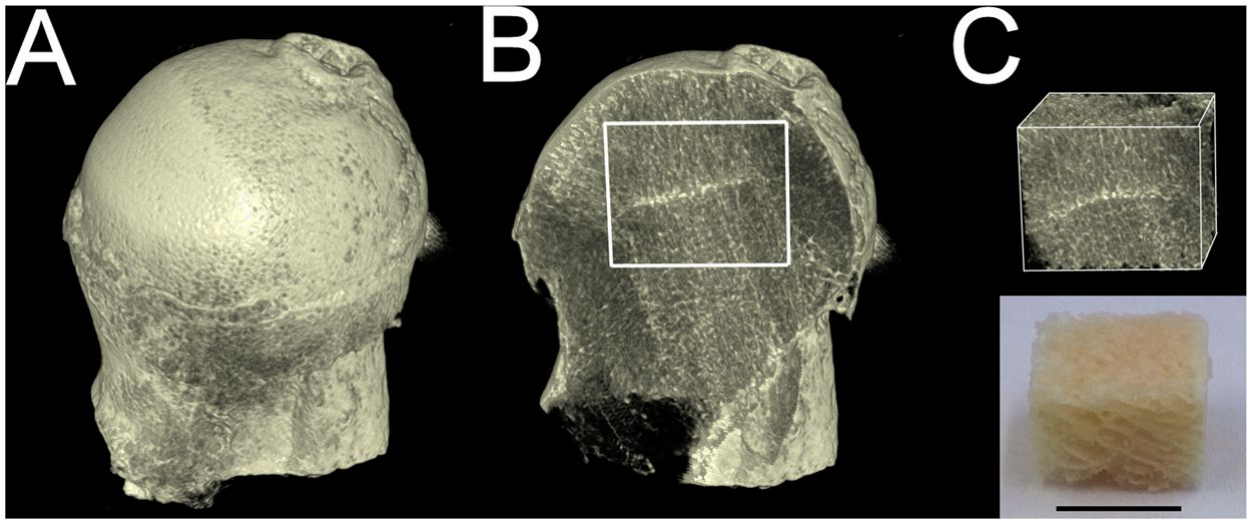
3D-reconstruction of a femoral bone sample showing the biopsy location. (**A**) Anterior view, (**B**) coronal section from the mid-plane with the biopsy site marked, (**C**) reconstruction of the bone biopsy and a photograph of the biopsy with bone marrow removed. Scale bar presents 1.0 cm.

The average age of female (n = 6) and male (n = 12) patients giving femoral samples was 63 years (range 50–79 years). Due to the anonymous nature of the Oulu University Hospital bone bank, no other information beside age and gender was known. A typical sample is shown in Fig. 1.

**Figure 2:**
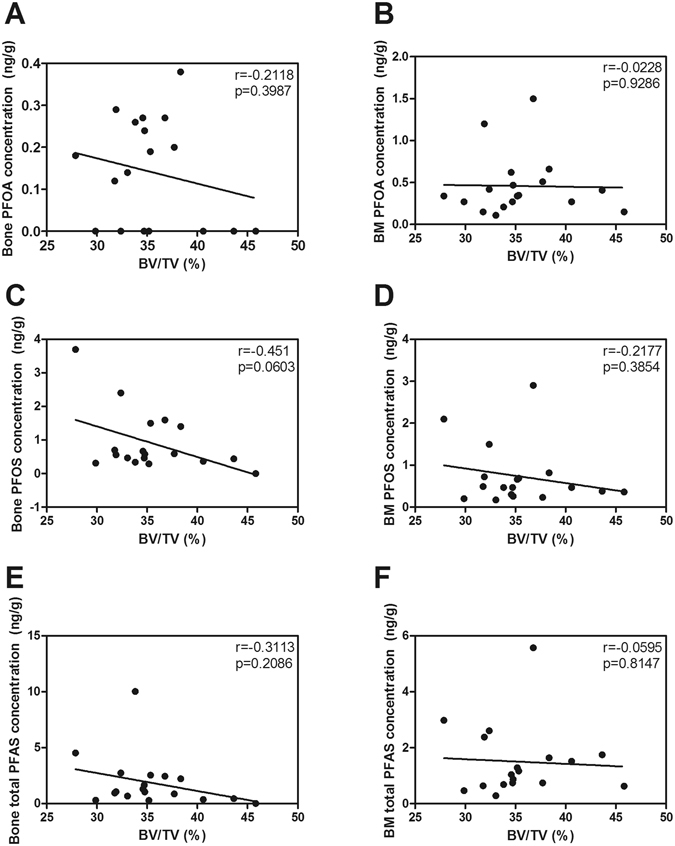
Dependence of bone volume/tissue volume (BV/TV) (**A**–**D**,**G**–**H**) and age (**E**–**F**) on concentrations of PFOA (**A**,**B**), PFOS (**C**,**D**) and total PFAS (**E**–**H**) in bone (left panel) and bone marrow (right panel). Linear correlations with r and p values.

**Figure 3:**
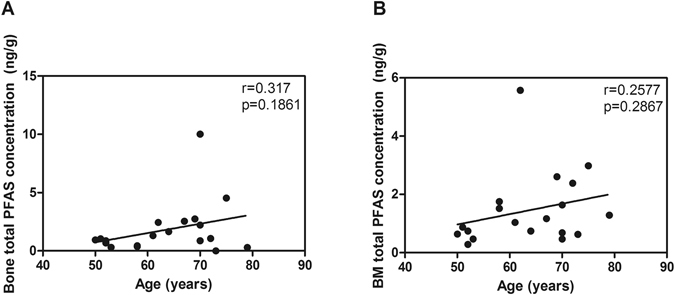
Dependence of age on total concentrations of PFAS in bone (**A**) and bone marrow (**B**).

A complete list of PFAS level in trabecular and bone marrow compartments and proportional bone volume [bone volume (BV)/tissue volume (TV)] percentage (BV/TV) are shown in Supplemental Table S1, and correlation between PFAS levels, proportional bone volume and age are shown in Figs 2 and 3, respectively. All the samples contained both PFOA and PFOS and other PFAS were detected in some of the samples. PFOA was prominent in bone marrow compared to trabecular bone, whereas PFOS was quite evenly distributed between bone marrow and trabecular bone. No significant linear correlations were observed between bone or bone marrow PFAS concentrations and age or proportional bone volume (Figs 2 and 3). However, there was a negative trend between bone PFOS concentration and relative bone volume (Fig. 2C, r = 0.45, p = 0.06).

### Cadaver biopsies

PFAS-concentrations from the tissue biopsies are listed in Table 1. The femur, tibia and fibula did not contain either PFOA or PFOS, but PFNA was present in all three long bones. The lung and liver contained the highest concentrations of PFAS, whereas bone marrow from the femur contained no PFAS.

**Table 1:** Biopsy concentrations of PFAS (ng/g ww) from the cadaver.

Sample	PFOA	PFNA	PFDA	PFUnA	PFDoA	PFHxS	PFOS
Bone							
Cranium	0.18	1.6	<LOQ^a^	<LOQ	<LOQ	<LOQ	0.17
Humerus	0.13	0.84	<LOQ	<LOQ	<LOQ	<LOQ	<LOQ
Rib	0.41	12	<LOQ	0.41	<LOQ	<LOQ	0.19
Femur, cortical	<LOQ	0.85	<LOQ	<LOQ	<LOQ	<LOQ	<LOQ
Femur, BM	<LOQ	<LOQ	<LOQ	<LOQ	<LOQ	<LOQ	<LOQ
Tibia, cortical	<LOQ	0.47	<LOQ	<LOQ	<LOQ	<LOQ	<LOQ
Tibia, BM	<LOQ	<LOQ	<LOQ	<LOQ	<LOQ	<LOQ	0.12
Fibula	<LOQ	0.19	<LOQ	<LOQ	<LOQ	<LOQ	<LOQ
Soft tissue							
Brain	0.2	<LOQ	<LOQ	<LOQ	<LOQ	<LOQ	0.37
Liver	0.7	0.73	0.6	0.78	0.3	<LOQ	4
Lung	0.66	0.87	0.56	0.67	<LOQ	0.14	6.7
Fat, subcutal	0.14	<LOQ	<LOQ	<LOQ	<LOQ	<LOQ	<LOQ

^a^Limit of Quantification.

### *In vitro* studies

#### Differentiation of osteoblasts

**Figure 4:**
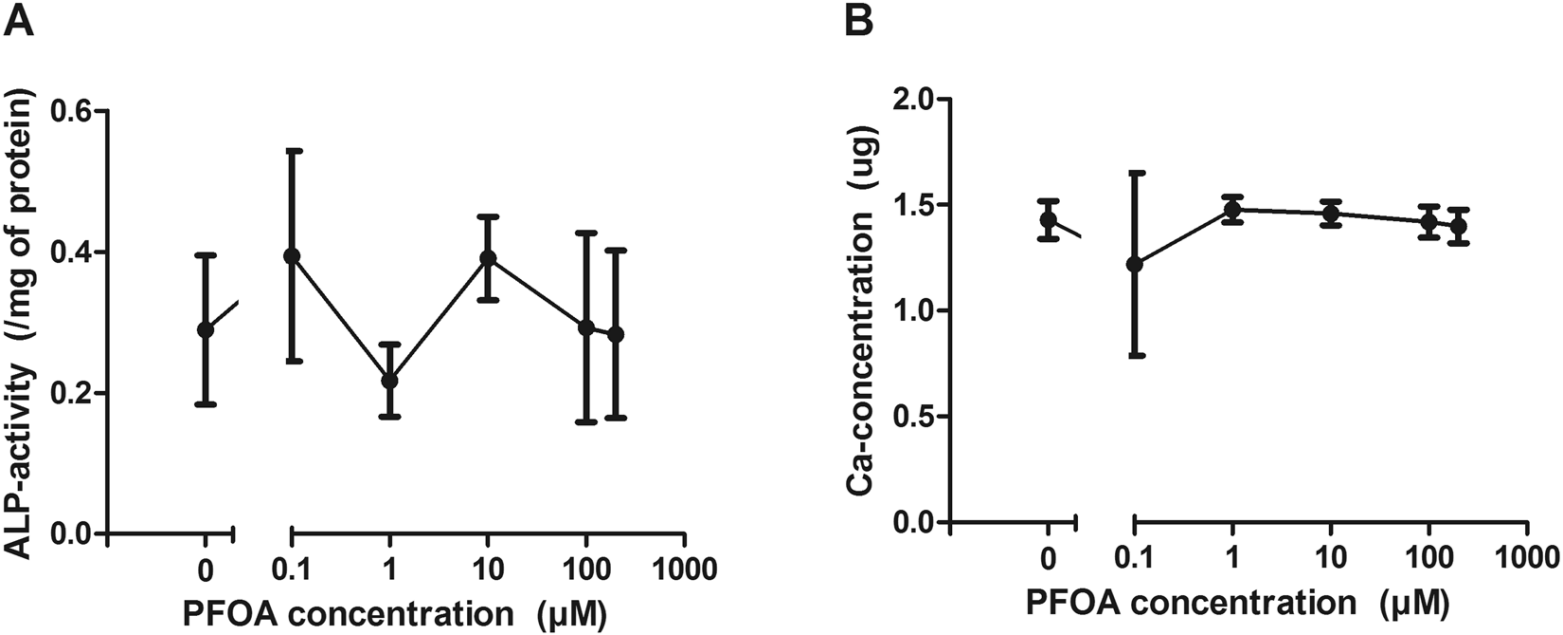
Effect of PFOA on ALP-activity (**A**) and calcium concentrations (**B**) in cultured human osteoblasts after exposure for 3 or 5 weeks. Plots represent mean ± SD.

The ALP-activity and the amount of calcium were measured from the differentiated osteoblasts after 3 and 5 weeks of differentiation, respectively. No significant differences were observed (Fig. 4A and B, respectively).

#### Differentiation of osteoclasts

**Figure 5:**
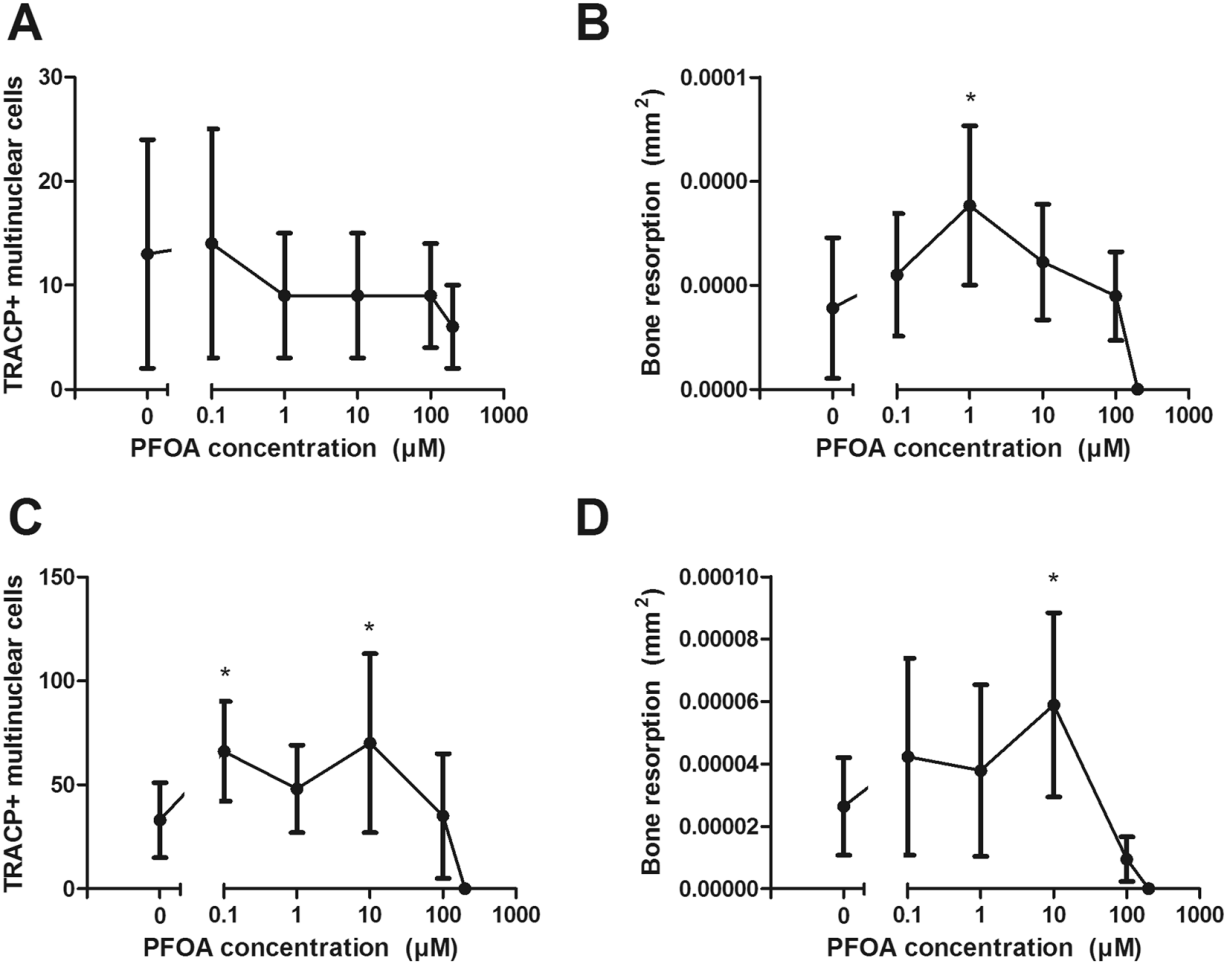
Effect of PFOA on the number of multinuclear TRACP + cells and resorption area in bone marrow (**A** and **B**) and peripheral blood derived osteoclasts (**C** and **D**) per bone slice after exposure for 10 days. Plots represent mean ± SD. *p < 0.05.

In bone marrow origin number of osteoclasts decreased non-significantly as the concentration of PFOA increased, whereas in peripheral blood origin the number increased and peaked significantly at 1.0 and 10 µM, and then dropped to zero at the highest concentration (200 µM). In both types of osteoclasts, the resorption area increased dose-dependently and peaked either at 1 µM or at 10 µM (Fig. 5).

In BMD calculations, the EC50-concentration for human osteoclast resorption activity was 0.49 µM, whereas the EC50 in mouse osteoclast resorption activity calculated from our previous study^15^, was 0.27 µM. Due to biological variation, no BMD could be reliably modeled from the number of osteoclasts in human, but in mice, the response was clearly more sensitive as the EC50 was 0.001 µM. For full data, please see Supplemental Table S2.

#### Bone effects in relation to PFOA concentrations

PFOA concentrations of human bones and mouse bones from our earlier study^15^ are summarized and compared to the lowest observed adverse effect levels (LOAEL) and no-observed adverse effect levels (NOAEL) from *in vivo* and *in vitro* studies in Table 2. For comparison, serum PFOA concentrations of general population and firefighters with potential occupational exposure are also shown. In our Finnish bone samples, the PFOA levels were about an order of magnitude below the levels of mouse bone samples associated with decreased bone mineral density and increased periosteal and medullary areas. However, the PFOA concentrations of the Spanish rib bone samples were about 400 times higher than in the Finnish trabecular bone samples, and also above the NOAEL of the *in vitro* studies with human osteoclasts. Although comparison between *in vivo* and *in vitro* concentrations is not straightforward, serum PFOA concentrations of general population and fire fighters were on average an order of magnitude below the NOAEL concentration in cell culture medium.

**Table 2:** Summary of PFOA concentrations (ng/g ww or ng/ml) in mouse and human bone, human serum samples and in *in vitro* studies of mouse and human osteoblasts and osteoclasts.

Study	n	% Detect	Mean	Median	Min-max	NOAEL	LOAEL
**Chemical analyses**							
Mouse bones							
Pooled femurs and tibias, controls^15^	2^a^	100	0.69	0.69	0.64–0.73	—	—
Pooled femurs and tibias, exposed^15^	2^a^	100	3.35	3.35	3.0–3.7	—	3.0^b^
Human bone bank (current study)							
Trabecular bone	19	63	0.15	0.18	<LOQ-0.38	—	—
Bone marrow	19	100	0.44	0.34	<LOQ-1.5	—	—
Other studies							
Spanish population, rib bone^12^	20	55	60.2	20.9	<LOQ-234	—	—
Swedish women, nursing, serum^41^	413	—	1.71	—	—	—	—
US Firefighters, serum^42^	12	100	7.0	6.0	2–12	—	—
US Population, serum^43^	2094	99.7	3.9	4.0	<LOQ-4.3	—	—
Finnish Firefighters, serum^44^	8	100	3.2	2.94	1.61–4.85	—	—
Swedish population, serum^45^	579	100	2.8	—	0.33–15	—	—
***In vitro –studies***							
Cells, mouse^15^							
Osteoblasts	—	—	—	—	—	41.4	414.1^c^
Osteoclasts	—	—	—	—	—	41.4	414.1^d^
Cells, human (present study)							
Osteoblasts	—	—	—	—	—	82 814^e^	—
Osteoclasts, PB	—	—	—	—	—	—	41.4^f^
Osteoclasts, MSCs	—	—	—	—	—	41.4	414.1^g^

Data from the present study and the indicated references. NOAEL = no-observed adverse effect level, LOAEL = lowest observed adverse effect level.

^a^Pooled samples from 20 animals, *n* = 5 in each group.

^b^Increased femoral periosteal and medullary area, decreased cortical mineral density of tibias.

^c^Increased osteocalcin expression.

^d^Increased resorption area.

^e^No significant effects.

^f^Increased number of osteoclasts.

^g^Increased resorption area.

## Discussion

Our aims were to investigate PFAS levels in human long bones and to study PFOA’s effects on human osteoblasts and osteoclasts. To our knowledge, this is the first study to focus solely on human bone as a target for PFAS and also to analyze the distribution of PFAS between bone and bone marrow. Due to anonymous nature of the bone bank material we did not have access to information on individual exposure histories, such as occupation or living area. We also had only one cadaver for studying the whole-body distribution of PFAS.

All femoral bone samples contained PFAS either in calcified bone itself and/or in bone marrow, and the most common and abundant compounds in the samples were PFOA, PFNA, and PFOS. When comparing the distribution of the substances between the two compartments, PFOA had higher concentrations in bone marrow than in trabecular bone, whereas PFOS was quite evenly distributed between bone marrow and trabecular bone. PFOA levels in this study were 7–8 times lower than in our previous study where exposure of pregnant mice led to altered bone geometry and decreased bone mineral density of the mouse offspring (Table 2). We observed no significant linear correlations between bone PFAS concentrations, age, and micro-CT-morphometrical parameters. However, the negative trend observed between bone PFOS concentration and relative bone volume (Fig. 2C) is in accordance with the earlier findings on a negative association between PFAS concentration and bone mineral density in the adult US population^13, 14^. PFOS exposure in the US population was much higher (mean serum PFOS 10.3 and 12.1 ng/ml in females and 15.1 and 19.2 ng/ml in males) compared to the levels in trabecular bone and bone marrow of the Finnish bone bank samples (min-max < LOQ-3.7 ng/g ww in trabecular bone and 0.17–2.9 ng/g ww in bone marrow), which may explain the lack of significant correlation in the Finnish samples.

Interesting is the high rib bone concentrations of PFAS’s in a Spanish population^12^ compared to our human and mouse data (Table 2). The difference might reflect a difference in background PFAS exposure between the Finnish and the Spanish populations. Contrary to many POPs, PFAS including PFOA and PFOS, are water soluble and can thus distribute to water resources, including ground water sources, and expose wild life and humans through drinking water. It has been reported that extensive use of fire-fighting foams has led to contamination of ground water supplies by PFOA and PFOS^24^. In Finland, water samples from five rivers contained 0.08–1.51 ng/l of PFOA and 0.13–8.95 ng/l of PFOS^25^, whereas in Spain, concentration means of 7.3–11.6 ng/l and 1.8–2.2 ng/l have been reported for PFOA and PFOS from rivers in highly populated areas, respectively^26^.

Our rib bone sample from the cadaver contained 0.41 ng/g ww of PFOA and 0.19 ng/g of PFOS, but neither was found from the femur, tibia or fibula of the cadaver. The liver and the lungs contained the highest amounts of PFAS, yielding a similar distribution pattern as previously reported^12^.

*In vitro* studies showed similar effects of PFOA on differentiation of human osteoclasts as in our previous study in mouse osteoclasts^15^. The studied concentrations include the range of levels analyzed in Spanish bone samples^12^, and are therefore environmentally relevant. Bone marrow derived precursor cells differentiated to osteoclasts showed a decreased trend in number, but significantly increased resorption activity, which peaked at 1 µM and then decreased as the concentration increased.

Osteoclasts differentiated from peripheral blood precursor cells significantly increased in number and in resorption activity up to 10 µM and then drastically decreased. The main difference between the two sources is the larger presence of stromal mesenchymal stem cells in the bone marrow sample, since not all the mesenchymal stem cells attach to the bottom of the flask, and thus they remain with the floating cells. In peripheral blood, stromal mesenchymal stem cells are also present, but to a much lesser degree. This can also explain the difference in responses to PFOA exposure seen in osteoclast number.

The possible mechanisms behind the increase in osteoclast number and resorption activity are unclear. However, PFOA might affect cytokine and clastokine profile during the differentiation process. PFOA has been shown to activate the complement system and especially the complement factor 3a (C3a) in C57BL/6 mice after 10-day oral exposure to a diet containing 0.002–0.02% w/w PFOA^27^. C3a is closely related to coupling of bone resorption by osteoclasts and bone anabolism by osteoblasts^28^. PFOA also suppresses β-catenin expression and down-regulates osteoclast-damping WNT-signaling in trophoblastic cells^29, 30^. In general, studies with knockout mice have indicated that the toxic effects of PFOA are mostly mediated via the peroxisome proliferator-activated receptor α (PPAR-α) subtype^9^. Both osteoclasts and osteoblasts express PPARs, which makes them potential targets for PPAR agonists^31, 32^. In addition, PFOA has been shown to be an agonist for CAR (Constitutive Androstane Receptor) and to induce the expression of CYP2B10 and CYP4A14 after heterodimerization with RXR (Retinoid X Receptor)^33, 34^. Similar to PPARs, CAR has been shown to regulate energy metabolism, and CAR-knockout male mice have increased bone mineral density compared to wildtype mice at least until 15 weeks of age^34^. In the same study, CAR was also shown to be expressed in both mesenchymal and osteoclastic cell lineages. In addition, strong CAR agonists, such as antiepileptic drugs phenytoin, phenobarbital and carbamazepine, have been associated with low bone mass and increased fracture risk, as reviewed by Vestergaard and colleagues^35^. It is therefore plausible that these nuclear receptor pathways are involved in the bone effects of PFOA.

According to BMD modelling of mouse and human data from our previous report and this study (EC50 0.27 vs. 0.49 µM, respectively), mouse osteoclasts seem to be slightly more sensitive to PFOA. Unfortunately, human data could not provide a reliable BMD for the number of osteoclasts, but in mice the EC50 for the number of cells was 0.001 µM, clearly more sensitive than the EC50 of resorption activity in mice. According to the data, PFOA the cell number is more sensitive to PFOA than the resorption activity of the cells.

In conclusion, PFAS are commonly present in human bone as they weredetected from every bone and bone marrow sample. Bone cell culture studies showed increased bone resorption activity at lower, partly environmentally relevant, concentrations in human-bone marrow and peripheral blood-derived osteoclasts, whereas no effect was observed in osteoblasts. There was no significant correlation between PFAS concentrations, age and bone properties. This study highlights the bone as potentially significant target tissue of PFAS toxicity and emphasizes the need for additional studies in order to clarify the significance of current population PFAS exposures on bone health.

## Materials and Methods

### Bone samples

Human femoral head samples (n = 18) were acquired from the clinical bone bank held in Oulu University Hospital, Oulu, Finland. The Special National Supervisory Authority for Welfare and Health (Valvira) granted permission for the use of the aged cadaver and live donor bone collection of clinically unusable specimens for research purposes (Decision 8.5.2009, Diary number 2240/05.01.00.06/2009). Only donor age and sex information were obtained from the tissue bank. Ten samples were scanned with cone beam computed tomography (CBCT) using a 451-row cone CT scanner (Scanora 3D, Soredex, PaloDEx Group Oy, Tuusula, Finland) with a field of view (FOV) of 60 mm × 60 mm, voxel size of 0.133 mm, and exposure time of 4.5 s. The parameters were a peak tube voltage of 85 kVp and a tube current of 15 mA. The captured images were reconstructed using a high-spatial frequency reconstruction algorithm. The reconstructed images were then re-oriented similarly and new cross-sectional images were produced with DataViewer software (Bruker MicroCT, Kontich, Belgium). The regions of interest (ROIs) were drawn for trabecular bone inside the femoral head, consisting of varying numbers of cross-sectional images depending on the sample size, and the threshold was optimized for trabecular bone, and analyzed with CTAn software (Bruker MicroCT, Kontich, Belgium). One sample was already cut in several pieces and therefore was left out of the morphometrical analyses.

Additional ten femoral head samples were scanned using the microCT device (Skyscan 1176, Bruker) inside a plastic container put into the chamber of the device to acquire more detailed data of the morphometrical properties of the bones. Two femoral heads were observed to have been sawed in half during the surgical hip-arthroplasty operations, and were excluded from the study. Projection images were acquired by scanning each sample with an image pixel size of 34.84 µm. X-rays were generated with a voltage of 65 kV and filtered with a 1.0 mm aluminum filter to reduce the beam hardening effect. One projection was collected every 0.7° over 360° with an exposure time of 100 ms. ROIs were drawn in the same manner as with the samples studied with CBCT described above. The BV/TV was in similar magnitude in both CBCT and microCT, and similar magnitude has also been observed in studies with human femoral heads and mandibles^36, 37^.

After imaging, samples were taken from the femoral heads with an electrical saw, as shown in Fig. 1. The cortical part of the bone was left out, so that the sample consisted only of trabecular bone and bone marrow. Bone marrow was then removed by centrifuging the sample at 1000 RPM for 5 min and taken as a sample. The residual bone marrow was then removed by applying hexane treatment and further centrifuging the sample at 300 RPM for 5 min, then incubating the sample for 1 h, and finally the sample was washed with 1xPBS. After drying in the laminar hood, the sample was powderized in a liquid nitrogen mill (Cryomill MM400, Retsch, Germany) for 2 min to avoid heating of the samples.

### Cadaver biopsies

Eight bone biopsies and five soft tissue biopsies were taken from a deceased 46-year-old female, who had died from kidney carcinoma. The deceased had donated her body to anatomic dissection and research (see above), and the samples were collected one week after the time of death. Bone samples were taken from the cranium, humerus, rib, femur, tibia and fibula, and soft tissue samples from the brain, liver, lung, and subcutaneous fat from thigh.

### Chemical analyses

All the samples of femoral head and cadaver tissue were pretreated for chemical analyses as described previously^15^. The concentration of 11 PFAS (PFOA, PFOS, perfluorononanoic acid (PFNA), perfluorodecanoic acid (PFDA), perfluoroundecanoic acid (PFUnA), perfluorododecanoic acid (PFDoA), perfluorotridecanoic acid (PFTrA), perfluorotetradecanoic acid (PFTeA), perfluorohexane sulfonate (PFHxS), perfluoroheptane sulfonate (PFHpS), and PFDS (perfluorodecane sulfonate) were analyzed with liquid chromatography negative ion electrospray tandem mass-spectrometry (LC-ESI-MS/MS) as described previously^38^. Levels of quantitation (LOQs) for PFAS were 0.1–0.3 ng/g.

### Human mesenchymal stem cell (hMSC) culture and osteoblast differentiation

Human bone marrow samples were collected from patients operated for hip osteoarthrosis. All patients gave their written informed consent according to the Declaration of Helsinki and the Ethical Committee of Oulu University Hospital had approved the study protocol. All the methods were performed according to the Declaration of Helsinki. Bone marrow samples were then used to acquire stromal mesenchymal stem cells to be differentiated into osteoblasts, and monocytes/precursor cells into human osteoclasts.

Medium containing 10 nM dexamethasone (Sigma-Aldrich), 10 mM β-glycerol phosphate (Sigma-Aldrich), and 50 μg/ml ascorbic acid (Sigma-Aldrich) in α-MEM (α-Minimum Essential Medium) was introduced to induce differentiation into osteoblasts. The next day, the medium was changed to exposure medium containing 0, 0.1, 1, 10, 100 or 200 μM PFOA (Sigma-Aldrich) dissolved in DMSO (dimethyl sulfoxide), or DMSO only in differentiation medium. The exposure medium was changed every 4th day and the cell morphology was observed daily under the microscope for confirming the normal cell morphology and monitoring the differentiation process towards an osteoblast lineage. During differentiation, cells started to secrete calcium that was detectable by microscopy after 5 days. MTT-tests (Methyl Thiazolyl Tetrazolium) were done 1, 3 and 5 weeks after the beginning of differentiation to assess the viability of the cells. After 3 weeks, ALP activity per milligram of protein was measured as described earlier^39^, and after 5 weeks, the degree of mineralization was measured using a Calcium Detection Kit (Abcam, Cambridge, MA) according to the manufacturer’s instructions.

### Human osteoclast culture

Human osteoclasts were differentiated from bone marrow and peripheral blood samples. Human bone marrow samples from the femoral collum and trochanteric region were taken during total hip arthroplasty from a 44-year old male and a 64-year old female, who had given permission to take and study the samples. The Ethical Committee of The Northern Ostrobothnia Hospital District approved the study. Approximately 5 ml of bone marrow was taken, and the bone marrow sample was first maintained in α-MEM (Corning Life Sciences, Tewksbury, MA) containing 10% FBS, 100 IU/ml penicillin and 100 µg/ml streptomycin and 24 mM Hepes buffer (Sigma-Aldrich, St. Louis, MO) at +37 °C (5% CO2, 95% air) for 1–2 days. After this pre-culture, the media containing the non-adherent mononuclear cells were collected and the isolation was continued with Ficoll-Paque Premium separation (GE Healthcare, Little Chalfont, UK) following the manufacturer’s protocol. Isolated monocytes were then seeded to 96-well plates (300000 cells/plate) with a 0.28 mm 2 human bone slice on the bottom of each well. Bone slices were sawed from tibias and femurs from the bone bank. The next day the differentiation medium containing RANKL 50 ng/ml, M-CSF 10 ng/ml, TGF-β 5 ng/ml and dexamethasone 1 µM was changed in the wells. The medium was changed every 3–4 days and the whole culture was maintained for 10 days.

A peripheral blood sample was taken from a healthy 45-year old volunteer male from the cubital vein. After the Ficoll-Paque treatment the isolated monocytes were then seeded in the same manner on 96-well plates containing bone slices, and the differentiation medium was changed the next day. The media for peripheral blood monocytes contained RANKL 20 ng/ml and M-CSF 10 ng/ml. The medium was changed every 3–4 days, and the culture was maintained for 10 days.

### Quantifying osteoclast differentiation

To evaluate the differentiation of hematopoietic precursor cells to osteoclasts, bone slices having osteoclasts were stained with a TRACP-kit (Sigma) at +37 °C according to the manufacturer’s instructions. The nuclei were stained with the DNA-binding Hoechst 33258 fluorochrome (Sigma) for 10 min at room temperature. TRACP-positive cells having three or more nuclei were considered as osteoclasts and were counted from bone slices using a fluorescence microscope (Zeiss AX10; Carl Zeiss Ltd., Hertfordshire, England) with a 20×/NA 0.5 objective (Zeiss).

After counting the osteoclasts, the cells were removed from the bone slice by gentle scraping and the slices were dyed with peroxidase-conjugated WGA-lectin (WGA; 20 µg/ml) for 30 min at room temperature. Slices were washed 3 times with PBS and counterstained using a diaminobenzidine (DAB) kit according to the manufacturer’s instructions (Invitrogen, CA, USA). The total area of resorption pits was measured by acquiring images of each bone slice with a fluorescence microscope (Zeiss AX10), 10×/NA 0.25 Objective (Zeiss) and QImaging Retiga 4000R camera with a digital image analyzer (MCID Core 7.0, Ontario, Canada). Ranges of interest (ROIs) were drawn for each resorption pit and analyzed with FIJI (ImageJ) software^40^.

### Statistics

All reported results represent means with standard error if not otherwise stated. In cell culture experiments, if the data were normally distributed, one-way ANOVA was used with the posthoc-test LSD. In a non-normally distributed data, Mann-Whitney U-test and Kruskal-Wallis H-tests were used. Pearson correlation was calculated for each specimen to compare PFAS concentrations against age and BV/TV. Dose responses were analyzed using the benchmark dose (BMD) method, PROAST version 38.9 in R software. Based on the likelihood ratio test, the best-fitted models were selected among the exponential model families. Benchmark doses (critical effect dose) and their lower and upper 95%-confidence bounds were calculated at a 50% change in response compared with unexposed control.

## Electronic supplementary material

Table S1

Table S2
