# Supplementary material for: Perfluoroalkyl substances in human bone: concentrations in bones and effects on bone cell differentiation
Source: Sci Rep. 2017 Jul 28;7:6841. doi: 10.1038/s41598-017-07359-6 (PMC5533791; doi:10.1038/s41598-017-07359-6)
Supplement: Supplementary file 1 — Table S1 [file 41598_2017_7359_MOESM1_ESM.doc]

**Perfluoroalkyl substances in human bone: concentrations in bones and effects on bone cell differentiation**

Koskela A^1*^, Koponen J^2^, Lehenkari P^1^, Viluksela M^2,3^, Korkalainen M^2^, Tuukkanen J^1^

^1^Institute of Cancer Research and Translational Medicine, Department of Anatomy and Cell Biology, Faculty of Medicine, University of Oulu, Oulu, Finland;

^2^National Institute for Health and Welfare, Chemicals and Health Unit, Kuopio, Finland;

^3^Department of Environmental and Biological Sciences, University of Eastern Finland, Kuopio, Finland

*Corresponding author: Antti Koskela (antti.koskela@oulu.fi), P.O.Box 5000, FIN-90014 University of Oulu, Oulu, Finland

**Table S1.** micro-CT-obtained relative bone volume percentage (BV/TV) and PFAS-concentrations (ng/g ww in bone marrow samples, ng/g dry weight in bone samples) from human femoral head samples (n=18). TRAB = Trabecular bone, BM = Bone marrow, <LOQ = below level of quantitation, F = female, M = male.

| Gender | Age | BV/TV | PFOA | |  | PFNA | |  | PFDA | |  | PFUnA | |  | PFHxS | |  | PFOS | |  |
| --- | --- | --- | --- | --- | --- | --- | --- | --- | --- | --- | --- | --- | --- | --- | --- | --- | --- | --- | --- | --- |
|  |  |  | TRAB | BM |  | TRAB | BM |  | TRAB | BM |  | TRAB | BM |  | TRAB | BM |  | TRAB | BM |  |
| F | 51 | 34.76 | 0.24 | 0.47 |  | 0.15 | 0.14 |  | <LOQ^1^ | <LOQ |  | 0.07 | <LOQ |  | <LOQ | <LOQ |  | 0.58 | 0.26 |  |
| F | 61 | 34.57 | 0.27 | 0.62 |  | 0.11 | <LOQ |  | 0.07 | <LOQ |  | 0.08 | <LOQ |  | 0.11 | 0.12 |  | 0.67 | 0.3 |  |
| F | 62 | 36.77 | 0.27 | 1.5 |  | 0.24 | 0.66 |  | 0.1 | 0.2 |  | 0.16 | 0.15 |  | 0.07 | 0.16 |  | 1.6 | 2.9 |  |
| F | 70 | 33.81 | 0.26 | 0.21 |  | 9.1 | <LOQ |  | <LOQ | <LOQ |  | 0.32 | <LOQ |  | <LOQ | <LOQ |  | 0.34 | 0.47 |  |
| F | 75 | 27.87 | 0.18 | 0.34 |  | 0.21 | 0.34 |  | 0.13 | <LOQ |  | 0.16 | <LOQ |  | 0.15 | 0.2 |  | 3.7 | 2.1 |  |
| F | 79 | 35.18 | <LOQ | 0.34 |  | <LOQ | <LOQ |  | <LOQ | <LOQ |  | <LOQ | <LOQ |  | <LOQ | 0.29 |  | 0.29 | 0.66 |  |
| M | 50 | 31.77 | 0.12 | 0.15 |  | 0.08 | <LOQ |  | <LOQ | <LOQ |  | <LOQ | <LOQ |  | 0.05 | <LOQ |  | 0.7 | 0.49 |  |
| M | 52 | 37.70 | 0.2 | 0.51 |  | 0.08 | <LOQ |  | <LOQ | <LOQ |  | <LOQ | <LOQ |  | <LOQ | <LOQ |  | 0.59 | 0.23 |  |
| M | 52 | 33.05 | 0.14 | 0.11 |  | 0.08 | <LOQ |  | <LOQ | <LOQ |  | <LOQ | <LOQ |  | <LOQ | <LOQ |  | 0.47 | 0.17 |  |
| M | 53 | 29.87 | <LOQ | 0.27 |  | <LOQ | <LOQ |  | <LOQ | <LOQ |  | <LOQ | <LOQ |  | <LOQ | <LOQ |  | 0.31 | 0.2 |  |
| M | 58 | 40.59 | <LOQ | 0.27 |  | <LOQ | 0.47 |  | <LOQ | 0.19 |  | <LOQ | 0.12 |  | <LOQ | <LOQ |  | 0.37 | 0.47 |  |
| M | 58 | 43.63 | <LOQ | 0.41 |  | <LOQ | 0.49 |  | <LOQ | 0.3 |  | <LOQ | 0.17 |  | <LOQ | <LOQ |  | 0.44 | 0.38 |  |
| M | 64 | 34.70 | <LOQ | 0.27 |  | 1.2 | <LOQ |  | <LOQ | <LOQ |  | <LOQ | <LOQ |  | <LOQ | <LOQ |  | 0.46 | 0.47 |  |
| M | 67 | 35.33 | 0.19 | 0.35 |  | 0.11 | 0.13 |  | 0.22 | <LOQ |  | 0.45 | <LOQ |  | 0.08 | <LOQ |  | 1.5 | 0.69 |  |
| M | 69 | 32.38 | <LOQ | 0.42 |  | 0.34 | 0.38 |  | <LOQ | 0.13 |  | <LOQ | <LOQ |  | <LOQ | 0.18 |  | 2.4 | 1.5 |  |
| M | 70 | 38.34 | 0.38 | 0.66 |  | 0.17 | 0.16 |  | 0.11 | <LOQ |  | 0.09 | <LOQ |  | 0.07 | <LOQ |  | 1.4 | 0.82 |  |
| M | 72 | 31.90 | 0.29 | 1.2 |  | 0.1 | 0.14 |  | <LOQ | <LOQ |  | <LOQ | <LOQ |  | 0.12 | 0.32 |  | 0.56 | 0.72 |  |
| M | 73 | 45.81 | <LOQ | 0.15 |  | <LOQ | 0.12 |  | <LOQ | <LOQ |  | <LOQ | <LOQ |  | <LOQ | <LOQ |  | <LOQ | 0.36 |  |
| Mean | 63 | 35.45 | 0.15 | 0.44 |  | 0.63 | 0.17 |  | 0.03 | 0.04 |  | 0.07 | 0.02 |  | 0.03 | 0.07 |  | 0.89 | 0.70 |  |
| Median | 63 | 34.73 | 0.18 | 0.34 |  | 0.10 | 0.13 |  | 0.00 | 0.00 |  | 0.00 | 0.00 |  | 0.00 | 0.00 |  | 0.56 | 0.47 |  |
| Min | 50 | 27.87 | <LOQ | 0.11 |  | <LOQ | <LOQ |  | <LOQ | <LOQ |  | <LOQ | <LOQ |  | <LOQ | <LOQ |  | <LOQ | 0.16 |  |
| Max | 79 | 45.81 | 0.38 | 1.5 |  | 9.10 | 0.66 |  | 0.22 | 0.30 |  | 0.45 | 0.17 |  | 0.15 | 0.32 |  | 3.70 | 2.90 |  |
| ^1^Limit of Quantification | | | | | | | | | | | | | | | | | | | | |
