# Supplementary material for: Perfluoroalkyl substances in human bone: concentrations in bones and effects on bone cell differentiation
Source: Sci Rep. 2017 Jul 28;7:6841. doi: 10.1038/s41598-017-07359-6 (PMC5533791; doi:10.1038/s41598-017-07359-6)
Supplement: Supplementary file 2 — Table S2 [file 41598_2017_7359_MOESM2_ESM.doc]

**Perfluoroalkyl substances in human bone: concentrations in bones and effects on bone cell differentiation**

Koskela A^1*^, Koponen J^2^, Lehenkari P^1^, Viluksela M^2,3^, Korkalainen M^2^, Tuukkanen J^1^

^1^Institute of Cancer Research and Translational Medicine, Department of Anatomy and Cell Biology, Faculty of Medicine, University of Oulu, Oulu, Finland;

^2^National Institute for Health and Welfare, Chemicals and Health Unit, Kuopio, Finland;

^3^Department of Environmental and Biological Sciences, University of Eastern Finland, Kuopio, Finland

*Corresponding author: Antti Koskela (antti.koskela@oulu.fi), P.O.Box 5000, FIN-90014 University of Oulu, Oulu, Finland

**Table S2.** Results of benchmark dose modeling showing significant dose-responses of resorption pit area and number of multinuclear cells after exposure to PFOA. BMDs (critical effect doses, CEDs) and their lower and upper bound confidence intervals (BMD-L and BMD-U) are shown for critical effect size (CES) of 50%.

| Cell source | Parameter | Model | BMD | BMD-L | BMD-U |
| --- | --- | --- | --- | --- | --- |
| Human | Resorption pit area | E2 | 0.49 | 0.31 | 1.18 |
|  | TRACP+ multinuclear cells | - | - | - | - |
| Mouse | Resorption pit area | E5 | 0.27 | 0.08 | 3.1 |
|  | TRACP+ multinuclear cells | E5 | 0.001 | 0 | 0.14 |
